# Differentiation of HL-60 cells into primed neutrophils for the evaluation of antiapoptotic effects of poorly soluble nanoparticles

**DOI:** 10.1371/journal.pone.0328717

**Published:** 2025-07-30

**Authors:** Tamara Hornstein, Klaus Unfried

**Affiliations:** IUF – Leibniz Research Institute for Environmental Medicine, Düsseldorf, Germany; West Virginia University School of Medicine, UNITED STATES OF AMERICA

## Abstract

**Background:**

Neutrophil apoptosis is an important determinant of intensity and duration of neutrophilic inflammation. The interaction of poorly soluble nanoparticles with primed neutrophils can reduce their natural apoptosis rates. This reaction may contribute to pathogenic consequences of increased neutrophilic inflammation. Toxicological studies aiming to identify hazards of such materials with primary neutrophils are however challenging due to the short life span of these cells and a high donor to donor variability. Our purpose was the establishment of a culturable neutrophil-like cell line as a suitable model for studies of antiapoptotic effects of poorly soluble combustion-derived environmental nanoparticles. Therefore, differentiation protocols for the myeloid HL-60 cell line based on commonly used differentiation inducers all-trans retinoic acid (ATRA) and dimethyl sulfoxide (DMSO) were established and compared.

**Results:**

The data demonstrate that only a combined cell treatment with ATRA and DMSO for a period of 5 days leads to the complete HL-60 differentiation with the typical neutrophil morphology and characteristic features of neutrophil maturation like cell cycle arrest, increase in differentiation marker CD11b, loss of proliferation marker CD71, and increased phagocytic capacity. Exposure of cells differentiated with ATRA + DMSO to carbon nanoparticles or proinflammatory cytokine granulocyte-macrophage colony-stimulating factor (GM-CSF) revealed a delay of apoptosis causally linked to intracellular reactive oxygen species (ROS). These data verified our earlier findings with human peripheral primed neutrophils from donors with slightly elevated proinflammatory blood plasma factors. Moreover, completely differentiated HL-60 cells possessed similar levels of L-selectin CD62L as neutrophils with primed immunophenotype, thus representing the biology of primed inflammatory neutrophils.

**Conclusion:**

Neutrophil-like HL-60 cells differentiated according to our protocol could be an appropriate substitute cell line model for studies on the effects of inhalable nanomaterials on primed inflammatory neutrophils like lung neutrophils. For such toxicological studies our cell model is preferable to peripheral neutrophils, as blood neutrophils not always occur in a primed state and primed lung neutrophils from donors are not available for this purpose.

## Introduction

Neutrophilic granulocytes represent the most abundant cell type of the innate immune system. After infection or tissue damage these cells act as key effector cells of inflammatory responses [1]. As neutrophils are terminally differentiated leukocytes, their lifespan in circulation is restricted to 8–20 h by constitutive programmed cell death. However, in the presence of proinflammatory factors neutrophil specific antiapoptotic mechanisms can delay apoptosis and life span can be prolonged up to a few days [2,3]. Therefore, regulation of life span and apoptosis have decisive impact on maintaining homeostasis of peripheral neutrophils under physiological conditions and also on degree and duration of neutrophil-dominated inflammatory reactions [4].

Xenobiotics that interfere with the regulation of neutrophil apoptosis in humans thus can be considered to have adverse effects on the functionality of the innate immune system and on duration and resolution of neutrophil-driven inflammation. In this context the inhalation of ultrafine particulate materials, which often cause inflammatory reactions in the airways, is of particular interest, as such materials might directly come into contact with alveolar neutrophils. A couple of reports describe effects of various nanoparticles on the viability and on apoptosis rates of neutrophils as significant key endpoints in the evaluation of particle toxicology. A whole series of nanomaterials (including TiO_2_, CeO, Pd, Au, Ag, Fe_3_O_4_, and ZnO) are known to delay apoptosis in neutrophils and are therefore considered to act as enhancers of inflammation [5–10]. However, there are also some reports about proapoptotic effects of nanoparticles (gold, silver, or PAA-coated iron oxide) in neutrophils and/or neutrophil-like differentiated HL-60 cells [11–16]. Other groups investigating the effects of silica nanoparticles or multi-walled carbon nanotubes describe a lack of effects on neutrophil viability [17,18].

In our own studies on the effects of carbon nanoparticles, as model particles for combustion-derived environmental nanoparticles, we observed delayed apoptosis in exposed human peripheral neutrophils from COPD patients and from healthy controls as well as in neutrophils from broncho-alveolar lavage of exposed animals [19,20]. More recent mechanistic studies employing a large donor sample group from healthy volunteers revealed that delayed apoptosis after carbon nanoparticle exposure was caused by the induction of intracellular reactive oxygen species (ROS). This reaction, however, was restricted to a subset of samples containing primed or preactivated neutrophils from donors with slightly elevated level of proinflammatory cytokines in blood plasma [21]. Such responding cells were characterized by a pattern of activation markers on their surface (high CD11b and low CD62L), which is known to be specific for lung neutrophils [22]. Our results suggest to investigate antiapoptotic effects of particulate materials in the airways by using cells representing the activation status of inflammatory tissue migrated neutrophils like lung neutrophils in order to identify possible hazards of this kind of exposure.

Controversial findings for the effects of nanomaterials on neutrophil life span in human samples and neutrophil-like differentiated HL-60 cells might be due to the missing validation of the test system for cellular priming and/or activation. The use of neutrophilic granulocytes from donors for estimating the antiapoptotic potential of inhalable materials has some major drawbacks like a high donor to donor variability, time- and cost-intense isolation protocols, and a possible affection of neutrophils by the isolation procedure. Against this background, our purpose was to establish a cell line-derived model of granulocytes representing primed inflammatory neutrophils by using the well-known myeloid HL-60 cell line [23,24]. By the use of various inducers, HL-60 can be differentiated into three distinct cell lineages (monocytic, eosinophilic, and neutrophilic). However, the cell line is mostly used for neutrophil differentiation [24].

A great deal of published work for neutrophil differentiation of HL-60 cells refers to treatments with different chemicals and factors like all-trans retinoic acid (ATRA), dimethyl sulfoxide (DMSO), granulocyte-macrophage colony-stimulating factor (GM-CSF), or dimethylformamide (DMF) used alone or in a combination for various time periods (1–9 days). Thus, it is common practice to differentiate such instable cell line as HL-60 with certain substances only for certain assays or for the specific applications [25–30]. For instance, ATRA or/and DMSO for a period up to 7 days were used for studies on apoptosis [31,32]. DMSO alone for 6 days was applied to differentiate HL-60 cells, which were proposed for the use as a model of inflammatory neutrophils such as bronchus neutrophils [33].

In the current study we describe the establishment of a reproducible, validated system of culturable, inflammatory neutrophilic granulocytes by HL-60 differentiation based on the most frequently used inducers ATRA and DMSO. The success of the treatment was monitored at different time points. Our requirements were that such cell model should match characteristics of primed neutrophils and should therefore be appropriate to study the antiapoptotic effects of nanoparticles. As reference material we used carbon nanoparticles (carbon black), which we consider as representative for the carbonaceous core of combustion-derived environmental nanoparticles. Such a standardized *in vitro* cell system might be useful for the identification of possible hazards of inhalable particulate materials, notably as lung neutrophils are not available for this kind of studies and peripheral neutrophils frequently occur in a non-primed state.

## Materials and methods

### Particles and particle suspensions

Carbon nanoparticles (CNP, Printex 90) with a primary diameter of 20 nm were purchased from Degussa (Germany). Particle suspensions were prepared in PBS as described [21,34]. Physico/chemical characterization of particles and particle suspensions were published earlier [21,34].

### Isolation of human neutrophilic granulocytes

Human peripheral blood neutrophilic granulocytes were isolated using a discontinuous Percoll gradient and subsequent hypotonic lysis of erythrocytes. Viability and purity of isolated neutrophils (≥95%) were assessed as described earlier [21,35].

### Differentiation of HL-60 cells

**Fig 1:**
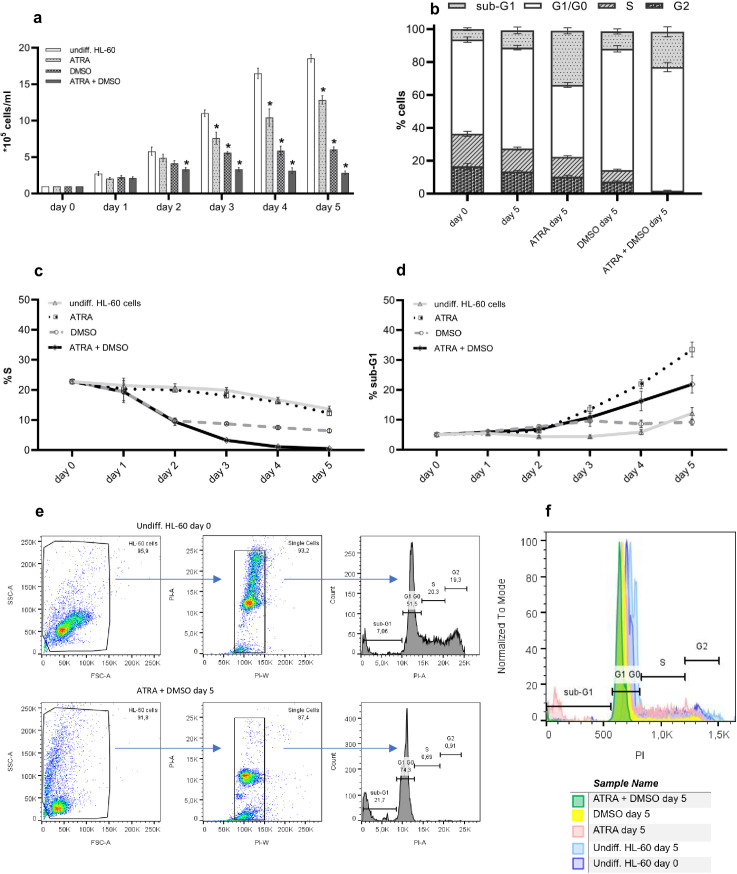
Effect of ATRA and DMSO on proliferation and cell cycle/apoptosis of HL-60 cells. a The time courses of HL-60 cell density after starting the differentiation with 1 µM ATRA or/and 1% DMSO, monitored for a period of 5 days. Data are presented as mean ± SEM, n ≥ 19, * p ≤ 0.05. **b** Distribution of the cell cycle phases (G0/G1, S, and G2) and apoptosis (sub-G1) after induction of differentiation with ATRA or/and DMSO before and after 5 days of treatment. Data are presented as mean ± SEM, n ≥ 9. **c**, **d** The time courses of S phase (%S) and apoptosis (%sub-G1) after starting the differentiation with ATRA or/and DMSO, monitored for a period of 5 days. Data are presented as mean ± SEM, n ≥ 15. **e** Representative gating strategy and histograms from typical flow cytometry measurement of the cell cycle. **f** Representative histogram for determination of DNA content and different cell cycle phases in undifferentiated and differently treated HL-60 cells.

The human myeloid HL-60 cell line (wild type CCL-240) was obtained from ATCC [23]. The cell line was cultured in suspension in RPMI 1640 medium containing 5% FCS (Biochrom) and 100 U/ml penicillin/100 µg/ml streptomycin (Sigma) at 37 °C in a humidified atmosphere with 5% CO_2_. As doubling time amounts to 36–48 h, cultivation was carried out by replacing the medium two to three times per week, maintaining the cell densities between 10^5^ and 10^6^ cells/ml. Differentiation was started 2–3 weeks after adaption of the cells to growth conditions and active cell growth was achieved with the doubling time of 24 h (Fig 1a). Cells were induced to differentiate into a neutrophil-like state by incubation at an initial density of 10^5^ cells/ml with cell culture medium supplemented with 1 µM all-trans retinoic acid (ATRA, Sigma) or/and 1% dimethyl sulfoxide (DMSO, Roth) for the indicated time spans.

### Cell growth

To analyze HL-60 proliferation, cells were counted microscopically at day 0 and on every day of the differentiation period using the trypan blue dye exclusion test.

### May-Gruenwald/Giemsa staining

Morphology of HL-60 cells was analyzed on cytospin slide preparations with 10^5^ cells in 500 µl PBS. After centrifugation (Cytospin3, Shandon) cells were air dried, stained with May-Gruenwald following by Giemsa staining (both from Roth), and studied using a Zeiss Axiophot light microscope. Obtained pictures were edited using the Zen 3.0 blue edition program (Zeiss).

### Phagocytosis test

Phagocytic capacity of HL-60 cells was analyzed as uptake of pH-sensitive fluorescence-labelled bioparticles (Thermo Fischer Scientific #35367) by flow cytometry. Particle fluorescence exclusively occurs after uptake into acidic phagosomes [30]. 1*10^6^ cells per sample were treated with 30 µg/ml of pHrodo Green (509/533 nm) labelled dead *Staphylococcus aureus* bioparticles for 1 h at 37 °C. Incubation at 4 °C was used here as a negative control to discriminate phagocytosis from passive particle uptake. Further controls were samples without pHrodo particles and pHrodo particles only. After incubation with bioparticles cells were washed once with PBS, stained with Dapi and analyzed by flow cytometry with FACSCanto II by use of FACSDiva 6.1.3 software (Becton Dickinson). After gating-out of residual debris, doublets, and dead cells, the percentage of pHrodo positive, phagocytic cells was estimated by analysis of the respective dot plot. 1*10^4^ events per sample were collected and analyzed. Graphics for publication were created by use of FlowJo 10.8.1 software (Becton Dickinson).

### Exposure of cells

HL-60 cells were seeded in 48-well plates at the density of 2*10^6^ cells in a volume of 1 ml in cell culture medium. For each method measurements of biological untreated controls at the particular time point were carried out. Carbon nanoparticle suspension was prepared freshly before use in PBS at stock concentration 1 mg/ml [21]. Granulocyte-macrophage colony-stimulating factor (GM-CSF, CST) was dissolved in distilled H_2_O at stock concentration 20 µg/ml and stored at −20 °C until used. Particles and GM-CSF were added to the seeded cells immediately and solvent controls were carried out.

### Analysis of cell cycle and apoptosis

For **cell cycle analysis**, HL-60 cells were stained according to Nicoletti protocol by direct DNA staining in propidium iodide (PI, Sigma) hypotonic solution and flow cytometry [36]. PI was dissolved in distilled H_2_O at 1 mg/ml and stored at 4 °C in the dark until used. 6*10^5^ cells per sample were suspended in a fluorochrome solution containing 0.1% sodium citrate (w/v), 0.1% Triton X-100 (v/v), and 50 µg/ml PI and incubated for 1 h at 4 °C in the dark. Thereafter cells were analyzed by flow cytometry and red fluorescence of PI (> 600 nm), bound to the DNA, was measured with FACSCanto II by use of FACSDiva 6.1.3 software (Becton Dickinson). After gate-out of residual debris and doublets the percentage of <2n hypodiploid DNA (%sub-G1, corresponding to fragmented DNA and thus to apoptotic nuclei), 2n diploid DNA (%G0/G1, corresponding to healthy cells/non-dividing cells), > 2n DNA (%S, corresponding to cells in the DNA replication phase), and 4n tetraploid DNA (%G2/M corresponding to mitotic cells) were estimated by analysis of the DNA histogram. 10^4^ events per sample were collected and analyzed. Graphics for publication were created by use of FlowJo 10.8.1 software (Becton Dickinson).

For **analysis of apoptosis**, cells were harvested after 18 h and apoptosis was assessed as described above according to Nicoletti protocol by the estimation of the percentage of apoptotic cells (%sub-G1 or %hypodiploidy) in the DNA histogram. Furthermore, apoptosis in HL-60 cells was verified with Annexin V-FITC/PI staining according to the instructions of the manufacturer (BioLegend #640914). Cells were harvested after 18 h and apoptosis was analyzed by the estimation of the percentage of total Annexin binding in the according histogram. 10^4^ events per sample were collected and analyzed.

### Measurement of intracellular ROS

HL-60 cells were harvested after 1 h and intracellular formation of ROS was assessed by 2’,7’-dichlorodihydrofluorescein diacetate staining (H_2_DCFDA, Santa Cruz) and flow cytometry. Non-fluorescent H_2_DCFDA was dissolved in DMSO at 100 mM and stored at −20 °C until used. 6*10^5^ cells per sample were resuspended in 0.2 mM H_2_DCFDA solution in PBS and incubated for 30 min at 37 °C in the dark. Thereafter cells were washed once with PBS and analyzed by flow cytometry. Green fluorescence of DCF (530 nm), corresponding to relative intracellular ROS formation, was measured in histogram (MFI statistic) with FACSCanto II by use of FACSDiva 6.1.3 software (Becton Dickinson) after elimination of residual debris and doublets. 10^4^ events per sample were collected and analyzed.

### Flow cytometric analysis of HL-60 cells and human peripheral neutrophils

Cells were isolated from the peripheral blood immediately after donation (neutrophils) or harvested at the respective day of the differentiation protocol (HL-60 cells) and were analyzed by staining of CD11b, CD71, CD16, CD32, CD64, CD66b, and CD62L cell surface markers and flow cytometry. First, 10^6^ cells per sample were incubated in Fc receptor blocking solution (#422302) to avoid non-specific binding of antibodies. Afterwards cells were directly incubated with fluorescently conjugated human antibodies CD11b-FITC (#301330), CD71-APC (#334108), CD16-APC (#302012), CD32-PE (#303206), CD64-PE (#305008), CD66b-PerCP/Cy5.5 (#305108), and CD62L-PE (#304806), or the corresponding volume of isotype matched antibodies FITC mouse IgG1 (#400109), APC mouse IgG2 (#400219), APC mouse IgG1 (400119), PE mouse IgG2b (400313), PerCP/Cy5.5 mouse IgM (401623), and PE mouse IgG1 (#400111) for 30 min at 4 °C in the dark. Fc receptor blocking solution and antibodies were purchased from BioLegend and used in the concentrations recommended by the manufacturer. Thereafter cells were washed once with PBS, stained with Dapi and analyzed by flow cytometry with FACSCanto II by use of FACSDiva 6.1.3 software (Becton Dickinson). After gating-out of residual debris, doublets, and dead cells the percentages of positive cells were estimated by analysis of the respective histograms. 2*10^4^ events per sample were collected and analyzed. Graphics for publication were created by use of FlowJo 10.8.1 software (Becton Dickinson).

### Statistical analysis

All data are presented as means ± SEM. Data analysis was performed with GraphPad Prism version 9.4.1 for Windows (GraphPad Software). Multiple comparisons were performed by applying one-way ANOVA with Dunnett’s post hoc tests for statistical significance. For two-factor analyses mixed effects models with Geisser-Greenhouse correction for matched measurement values were applied. Post hoc analyses (Dunnett’s or Tukey’s test) were performed in order to determine differences between groups. Analysis for significance between two groups were paired or unpaired Student’s t-tests (normally distributed values). In case of non-normally distributed results or small sample size, ranked tests were applied (Wilcoxon matched pairs test or Mann-Whitney ranked test). Data were considered to be statistically significant when p ≤ 0.05.

## Results and discussion

### Neutrophil differentiation of HL-60 cells

In order to develop a robust and reproducible neutrophilic cell model to determine the antiapoptotic effect of inhalable materials, we investigated the impact of ATRA and DMSO each alone or in combination at different time points of treatment. The success of the differentiation was tested by monitoring of cell morphology and characteristic changes of neutrophil maturation like proliferation and cell cycle, apoptosis, phagocytic capacity, as well as cell surface markers of differentiation and priming.

As terminally differentiated cells, neutrophils are unable to enter S-phase of the cell cycle and to further divide. We therefore first checked which treatment most efficiently leads to growth arrest and an apoptosis rate, which is comparable to that of freshly isolated human neutrophils. At the level of cell division, the combination of differentiation inducers ATRA and DMSO affected the proliferating ability of HL-60 cells most efficiently (Fig 1a). The analysis of the cell cycle by measurement of the DNA content (Fig 1e and 1f) showed that this loss of proliferative capacity is mostly caused by an accumulation of the cells in the G0 resting phase after 5 days (Fig 1b). A single treatment with ATRA or DMSO alone for 5 days did not cause an entire cell cycle arrest (Fig 1b and 1c). The effect of ATRA was limited to markedly increased apoptosis from day 2 of the treatment (Fig 1d). DMSO caused only partial differentiation as a decrease in S-phase occurred at day 2 after treatment, though with a plateau phase and without entire inhibition of proliferation (Fig 1b and 1c). Increased apoptosis rates and the lack of cells in S and G2 phases after 5 days of ATRA + DMSO treatment confirmed the complete terminal differentiation achieved by this treatment protocol (Fig 1b - 1d).

**Fig 2:**
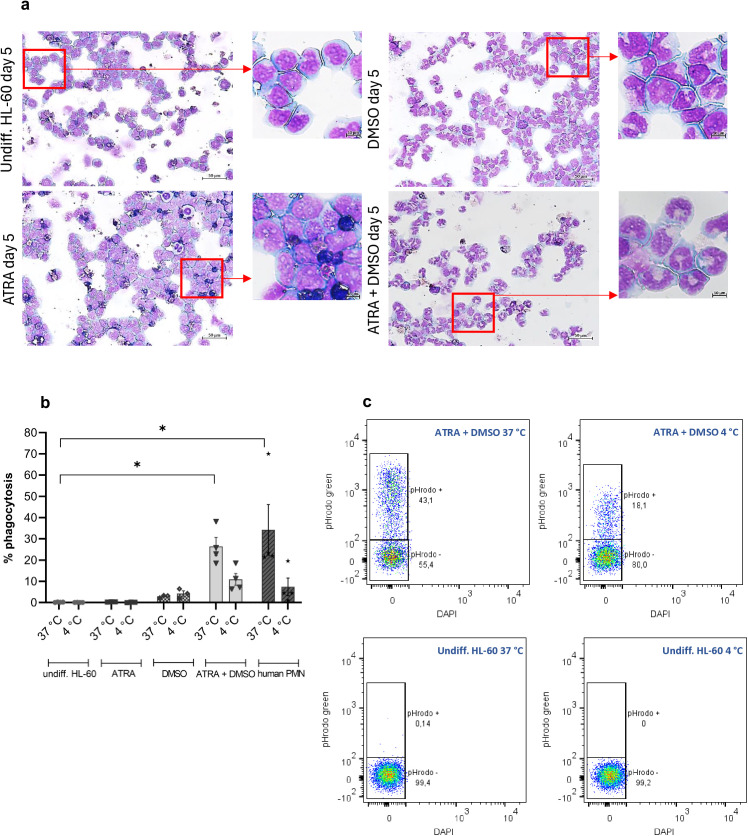
Effect of ATRA and DMSO on morphology and phagocytic capacity of HL-60 cells. a HL-60 cells were treated with 1 µM ATRA and 1% DMSO alone and in combination for 5 days and morphology was assessed after May-Gruenwald-Giemsa staining. Representative images are shown. **b** Phagocytic capacity of undifferentiated, differently treated HL-60 cells at day 5 of differentiation protocol, and freshly isolated human peripheral blood neutrophils (PMN). Data are presented as mean ± SEM, n ≥ 3, * p ≤ 0.05. **c** Representative dot plots from typical flow cytometry measurement for determination of pHrodo positive, phagocytic cells.

After 5 days of treatment with a combination of the inducers ATRA and DMSO HL-60 cells showed typical neutrophil morphology like decreased nucleus/cytoplasm ratio, kidney-like or segmented nuclei, and less basophil cytoplasm, compared with undifferentiated cells (Fig 2a). ATRA alone did not change the HL-60 morphology, but increased the numbers of dead cells. DMSO caused incomplete differentiation with visibly diminished numbers of apoptotic cells, confirming the data from PI staining (Fig 1b, 1d and 2a). Undifferentiated HL-60 after 5 days in culture remained as typical promyelocytic cells with large round nuclei and basophil cytoplasm, confirming the absence of significant level of spontaneous differentiation as previously observed by the cell cycle analysis (Fig 1b and 2a).

As a typical feature of neutrophilic granulocytes phagocytosis was determined in cells differentiated under the different regimen. The uptake of pHrodo Green labelled *S. aureus* bioparticles was measured by flow cytometry. Undifferentiated and ATRA-treated HL-60 cells possessed no phagocytic capacity, and DMSO addition resulted in only slight increase of numbers of phagocytic cells (3.3%) (Fig 2b). Combined treatment of HL-60 with ATRA and DMSO for 5 days led to a markedly higher percentage of pHrodo phagocytosing cells (26%) and it was comparable with the level of phagocytosis observed for human blood neutrophils (Fig 2b and 2c).

**Fig 3:**
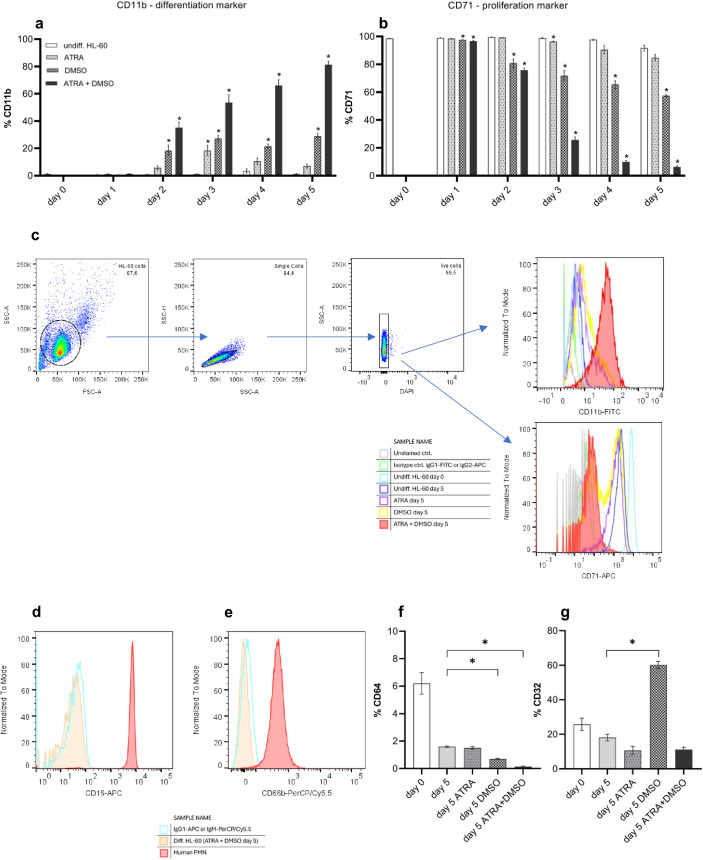
Changes in cell surface markers upon differentiation of HL-60 cells. **a**, **b** The time courses of CD11b and CD71 positive cells after starting the differentiation with 1 µM ATRA or/and 1% DMSO, monitored for a period of 5 days. **c** Representative gating strategy and histograms from typical flow cytometry measurements for determination of CD11b/CD71 positive HL-60 cells. **d**, **e** Representative histograms from typical flow cytometry measurements for determination of CD16 (FcγRIII) and CD66b expression in HL-60 cells and freshly isolated human peripheral blood neutrophils (PMN). **f**, **g** Changes in CD64 (FcγRI) and CD32 (FcγRII) positive cells after induction of differentiation with ATRA or/and DMSO before and after 5 days of treatment. Data are presented as mean ± SEM, n ≥ 3, * p ≤ 0.05.

Neutrophil-like differentiation was also monitored at the level of typical cell surface markers CD11b and CD71. CD11b as α_M_ chain of the integrin CD11b/CD18 and complement receptor CR3 is almost absent on exponentially proliferating HL-60 cells, and therefore is associated with phagocytic capacity and neutrophil differentiation [29,37]. The transferrin receptor 1 (CD71) is found on all proliferating cells and is used as a marker of undifferentiated HL-60 cells [38]. Among CD71 positive myeloid HL-60 (day 0) only a very low percentage of cells labelled with CD11b antibody could be detected. The level of spontaneous differentiation remained minimal as no significant changes in CD71 and CD11b were observed after 5 days of cultivation (Fig 3a and 3b). The effect of ATRA or DMSO alone on surface marker appearance was insufficient. ATRA caused a significant increase of CD11b expression at day 3 (18.4%), decline up to 7.2% of CD11b positive differentiated cells at day 5 of treatment, and coincident high level (85%) of CD71 positive, still proliferating cells 5 days after incubation (Fig 3a-c). DMSO led to distinct differences in surface markers already at day 2 and induced, compared with ATRA, higher, but incomplete level of differentiation with 28.9% of CD11b and 57.4% of CD71 positive cells at day 5 of differentiation protocol (Fig 3a-c). However, ATRA and DMSO in combination showed again an obvious synergistic effect on neutrophil differentiation from day 2 after treatment and yielded a population with 81.4% CD11b and 6% CD71 positivity at day 5 (Fig 3a-c).

Additionally, FcγRIII (CD16) and CEACAM-8 (CD66b) as well-known indicators for primary neutrophilic granulocytes were monitored here as additional markers for neutrophil differentiation. However, we could not observe CD66b and CD16 cell surface expression in HL-60 cells during the whole differentiation protocol or in untreated cells. Human peripheral blood neutrophils served as a positive control in these experiments (Fig 3d and 3e). The data in the literature concerning CD16/CD66b expression in HL-60 cells is conflicting, with reports about absence, but also induced positive expression during the differentiation [39–42]. Analysis of further Fcγ receptors CD64 (FcγRI) and CD32 (FcγRII) revealed positive expression in undifferentiated HL-60 cells (6% and 26%) (Fig 3f and 3g). Treatment of HL-60 with ATRA and DMSO alone caused decline in CD64 expression, and a combination of both differentiation inducers caused complete degression at day 5 of incubation (fig 3f). ATRA and ATRA + DMSO together caused decrease in CD32 expression up to 11% at day 5 of differentiation protocol, and only DMSO exposure significantly induced CD32 levels up to 60% at day 5 of treatment (Fig 3g). Most often CD64/CD32 expression was reported in the literature to be sustained high [43,44] or induced upon HL-60 differentiation [39,41,44,45]. No expression of CD64 was observed in un- and DMSO-differentiated HL-60 cells after 5 days of treatment [41].

Despite the absence (for CD16 and CD64) or quite low expression (11% positivity for CD32) of opsonic phagocytic Fcγ receptors, the phagocytosis level of ATRA + DMSO differentiated HL-60 was comparable with the phagocytic ability of human primary neutrophils, that are known to be Fcγ receptor positive (Fig 2b and 3d). Moreover, increased phagocytosis in ATRA + DMSO differentiated HL-60 cells was associated with increased surface expression of α_M_ chain (CD11b) of complement receptor CR3 (Fig 3a). Low numbers of CD11b positive cells in ATRA differentiated samples (7.2%) somehow mirror no phagocytic ability of these cells at day 5 of treatment (Fig 2b and 3a). Moreover, 5-days DMSO differentiated HL-60 cells possess higher CD11b levels (28.9%) and 60% CD32 positivity, but still extremely low (3.3%) phagocytic function (Fig 2b, 3a and 3g). Thus, complement receptors like CR3 (containing α chain CD11b) and complement-dependent pathway seem to play a crucial role in the phagocytosis in HL-60 cells and neutrophils as Fc receptors [46,47]. High CD11b positivity, together with a significant decline of proliferation marker CD71, and a significantly increased phagocytic function, compared with the undifferentiated cells (Fig 2b, 3a and 3b), allow us to consider HL-60, differentiated with ATRA + DMSO for 5 days, as a fully differentiated neutrophil-like cell line with phagocytic function, that also could be used as a simple cell model for opsonophagocytosis assays (OPA).

**Fig 4:**
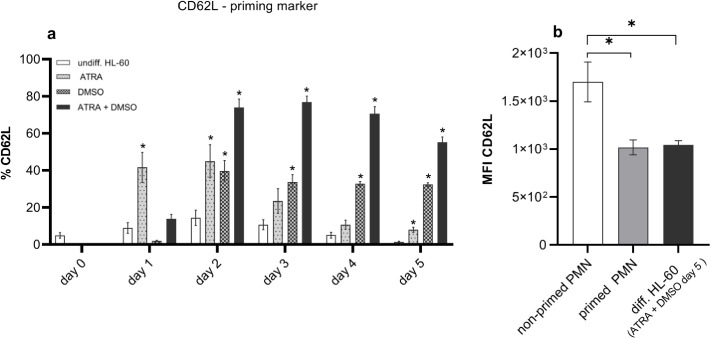
Changes in CD62L antigen upon differentiation of HL-60 cells. **a** The time courses of CD62L positive cells after starting the differentiation with 1 µM ATRA or/and 1% DMSO, monitored for a period of 5 days. **b** CD62L staining on freshly isolated human peripheral blood neutrophils (PMN) and fully differentiated neutrophil-like HL-60 cells (combined treatment ATRA + DMSO day 5). The data from human neutrophils (see Fig 4b) are originated from the earlier publication [21] in order to demonstrate the similarity of L-selectin levels in human primed responder neutrophils and differentiated cultured HL-60 cells. Data are presented as mean ± SEM. **a** n ≥ 3, **b** primed PMN n = 42, non-primed PMN n = 12, diff. HL-60 cells n = 11. * p ≤ 0.05.

In our earlier studies we observed that only primed or preactivated human peripheral neutrophils from donors with elevated proinflammatory blood plasma cytokine levels, and which were characterized by reduced levels of L-selectin CD62L, were able to respond on carbon nanoparticle exposure by delayed apoptosis. We termed such sets of cells “responder” cells, whereas naïve non-primed neutrophils with unaffected life span following carbon nanoparticle exposure were classified as “non-responder” neutrophils [21]. Moreover, the primed immunophenotype of decreased CD62L together with enhanced CD11b is well-known from airway neutrophils as well as from blood neutrophils of patients suffering from airway diseases [22]. As described above, increased CD11b levels are also considered as differentiation marker most efficiently induced by ATRA + DMSO up to 80–90%. As second marker for neutrophil priming, we investigated CD62L levels on HL-60 cells treated with ATRA or/and DMSO for a period of 5 days. The level of CD62L on undifferentiated HL-60 myeloid cells was very low during the whole time span of the differentiation protocol (Fig 4a). ATRA alone affected CD62L appearance markedly already after 24 h treatment with a peak at day 2 (45%) and a decline up to 8% at day 5 of differentiation. DMSO alone caused a similar increase in CD62L level 2 days after treatment (40%) with a following plateau phase after day 3 (Fig 4a). Combined treatment of ATRA and DMSO induced a synergistic effect with a steep early increase between day 1 and day 2, a maximum at day 3 (77%), and a marked decline up to 55.2% until day 5 (Fig 4a). In order to estimate whether the level of CD62L achieved by the combined treatment of HL-60 cells is comparable to primed neutrophils, which respond by a reduction of apoptosis rates, we also analyzed the fluorescence intensity of CD62L staining in peripheral neutrophils from responding and non-responding samples. Comparing ATRA + DMSO-differentiated HL-60 cells with human peripheral neutrophils, we observed that primed responder neutrophils display approximately the same L-selectin levels. Naïve, non-responding cells however bear significantly higher levels of CD62L on their surface (Fig 4b). Together with our findings on CD11b, we consider this result as a strong indication, that our differentiation protocol produces cells representing human neutrophils, which are in a state of priming or activation comparable to inflammatory migrated tissue neutrophils like lung neutrophils.

### Delayed apoptosis in differentiated HL-60 cells exposed to external stimuli

The regulation of delayed neutrophil apoptosis is closely linked to the oxidant status of the cell [48]. ROS, as second messengers, are known to trigger antiapoptotic signaling pathways. Earlier studies on the effect of the proinflammatory cytokine granulocyte-macrophage colony-stimulating factor (GM-CSF) on neutrophils demonstrated, that GM-CSF extends the life span of neutrophils via modulation of oxidant dependent PI3-K signaling and inhibiting cell apoptosis [49,50]. However, for some nanoparticles antiapoptotic effect without a detectable increase of ROS in neutrophils was reported [6,10]. In our own work we were able to show that exposure to carbon nanoparticles leads to an increase of intracellular ROS in human neutrophils causing reduced apoptosis rates [21]. Therefore, the ability to increase intracellular ROS after exposure to external stimuli appears to be relevant for the success of our differentiation protocol.

Since we aimed to verify our findings with primed human neutrophils on carbon nanoparticle-induced antiapoptotic and prooxidative effects in neutrophil-like HL-60 cells, we tested the ability to increase ROS levels and the subsequent causally linked delay of apoptosis after giving external stimuli. Thus, undifferentiated controls and differently treated HL-60 cells were exposed to 20 ng/ml GM-CSF, that is also known to cause increased intracellular ROS and delayed apoptosis, or 33 µg/ml of carbon nanoparticles (carbon black with a primary diameter of 20 nm). The exposure dose of carbon nanoparticles was chosen as it proved to be an effective dose in earlier experiments, representing a cumulative exposure to environmental air pollution [19,20]. Dose response experiments showed, that primed neutrophils also respond to much lower particle doses, probably representing short term or single exposure by increased intracellular ROS and reduced apoptosis rates [21].

**Fig 5:**
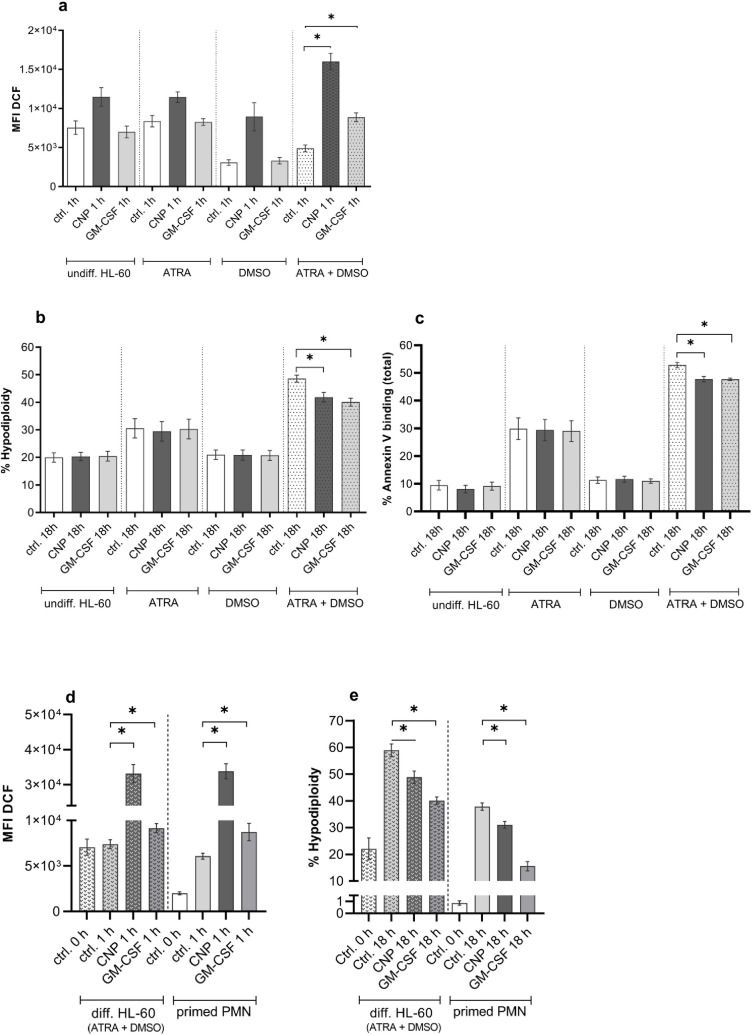
Effect of granulocyte-macrophage colony-stimulating factor (GM-CSF) and carbon nanoparticles (CNP) on apoptosis and ROS production in HL-60 cells. Undifferentiated and differently treated HL-60 cells (day 5) or freshly isolated human peripheral blood neutrophils (PMN) at the density of 2*10^6^ cells/ml were exposed to 20 ng/ml GM-CSF or 33 µg/ml CNP. Controls were cells before treatment (ctrl. 0 h) and incubated untreated cells (ctrl. 1 h/18 h). **a, d** DCF fluorescence determined at 1 h after exposure. **b, e** Apoptosis (%hypodiploidy or %sub-G1) determined at 18 h after exposure. **c** Apoptosis (% total Annexin binding) determined at 18 h after exposure. The data from human neutrophils (see Fig 5d and 5e) are originated from the earlier publication [21] in order to demonstrate the similarity of ROS levels and apoptosis behaviour in response to proinflammatory cytokine GM-CSF and carbon nanoparticles in human primed responder neutrophils and differentiated cultured HL-60 cells. Data are presented as mean ± SEM. **a** n ≥ 4, **b** n = 4, **c** n = 6, **d** PMN n = 77, diff. HL-60 cells n = 44, **e** PMN n = 123, diff. HL-60 cells n = 42. * p ≤ 0.05.

The generation of intracellular ROS was determined 1 h after exposure by H_2_DCFDA fluorescence assay (Fig 5a). GM-CSF was able to enhance the level of intracellular ROS significantly only in neutrophil-like cells differentiated with ATRA and DMSO. By contrast, the effective dose of 33 µg/ml carbon nanoparticles led to a significant increase in intracellular ROS in un- and differentiated cells, however with a trend to stronger ROS formation in neutrophil-like cells differentiated via combined treatment (Fig 5a).

Apoptosis rates were determined 18 h after exposure by determination of cells with subG1 status according to Nicoletti [36]. As expected from the data of cell cycle analysis, compared with control cells, spontaneous apoptosis rates were higher in ATRA and ATRA + DMSO treated cells, while in ATRA + DMSO-differentiated HL-60 apoptosis was markedly elevated, approximately reflecting the natural apoptosis rates of isolated human peripheral neutrophils (Fig 5b). GM-CSF and carbon nanoparticle treatment had no effect on apoptosis rates in undifferentiated and in ATRA- or DMSO-treated HL-60 cells. In ATRA + DMSO completely differentiated neutrophil-like cells a significant decrease of apoptosis was observed (Fig 5b). The validity of this finding was corroborated by Annexin V/PI staining as a second, independent method (Fig 5c).

Prooxidative and antiapoptotic effects of carbon nanoparticles, observed in fully differentiated neutrophil-like HL-60 cells, nicely reflect our earlier findings with human peripheral neutrophils primed by a mild proinflammatory environment in host blood plasma and termed “responder neutrophils” based on particle-induced antiapoptotic effect (Fig 5d and 5e) [21]. The causal link of increased ROS to reduced apoptosis rates following carbon nanoparticle exposure, that we earlier confirmed by antioxidant strategies in primed responder neutrophils and in differentiated HL-60 cells, could only be reproduced in cells differentiated by ATRA and DMSO. This result obviously matches the finding, that in non-primed neutrophils with high CD62L, although showing high intracellular oxidant capacity, no change in apoptosis rates occurred after particle exposure [21]. Primed neutrophils exhibited similar features (like decreased levels of CD62L and delayed apoptosis following particle exposure) as HL-60 cells, differentiated for 5 days with ATRA and DMSO (Fig 4b and 5e).

Thus, responder neutrophils, as well as HL-60 cells differentiated with DMSO and ATRA according to our protocol, not just share a mature primed immunophenotype but also possess a similar pattern and strength of response to proinflammatory cytokine GM-CSF and carbon nanoparticles. Moreover, carbon nanoparticle-induced antiapoptotic effect has been also observed in human neutrophils from COPD patients [20]. These data suggest, that primed neutrophils and ATRA + DMSO-treated completely differentiated neutrophil-like HL-60 cells may represent inflammatory neutrophils like lung neutrophils, that have the ability to exacerbate airway inflammation by prolonging their life span [22].

The HL-60 cell line has been used as a model of human neutrophils in a couple of toxicological studies, which, however differ markedly in their differentiation strategy. In some of these studies outcomes of exposure appeared similar in peripheral neutrophils and in the respective differentiated HL-60 cells. Similar patterns of inflammatory response (reactive oxygen species, extracellular traps, and cytokine release) have been observed in HL-60 differentiated by DMSO alone and in human primary neutrophils following exposure to Ag, CuO or TiO_2_ nanoparticles [51]. Another study, in which cytotoxicity was determined in ATRA-differentiated HL-60 cells and in human primary neutrophils after exposure to carbon nanotubes, describes the absence of effects on both cell types [17]. Both groups conceded, that HL-60 cells are not the perfect substitute for blood neutrophils and recommended HL-60 cells rather for the initial screening of nanomaterial-induced inflammation. In another approach undifferentiated HL-60 cells were observed to be a suitable model for studies of oxidative stress, toxicity, and apoptosis/necrosis after combined exposure to two common environmental pollutants like carbon black particles and electromagnetic radiation [52]. These studies aim to validate their results by the similarity with the findings from exposed blood neutrophils. However, in our earlier studies we describe, that blood neutrophils exhibit a high donor to donor variability with respect to effects on apoptosis rates.

In our opinion, 5-days HL-60 cells differentiated with combined treatment of ATRA + DMSO may to be a substitute for blood neutrophils particularly for examination of apoptosis rates. We therefore suggest HL-60 cells, differentiated according to our protocol, for use as a robust model for primed inflammatory neutrophilic granulocytes for studies of antiapoptotic, inflammation enhancing effects of particulate materials like carbon nanoparticles and maybe other poorly soluble combustion-derived environmental nanoparticles.

## Conclusion

The data presented here demonstrate that human myeloid HL-60 cell line exposed to a combined treatment of ATRA and DMSO for a period of 5 days exhibit typical neutrophil morphology and characteristic features of neutrophil maturation like cell cycle arrest, increase in differentiation marker CD11b, loss of proliferation molecule CD71, and increased phagocytic capacity.

Formation of intracellular ROS and the apoptotic course after exposure of fully differentiated neutrophil-like HL-60 cells to the proinflammatory cytokine GM-CSF or carbon nanoparticles were similar to the observed effects in human peripheral neutrophils. Moreover, acquired lower levels of the adhesion and priming marker CD62L and carbon nanoparticle-mediated prolongation of the life span in neutrophil-like HL-60 cells reflect obviously the behaviour of human primed peripheral neutrophils from donors with a slight elevated proinflammatory environment, thus representing the biology of primed inflammatory neutrophils. Neutrophil-like cells derived from the HL-60 cell line according to the described protocol could be an appropriate model for studies on the effects of inhaled nanomaterials on migrated inflammatory neutrophils like lung neutrophils. Due to high donor to donor variation, peripheral neutrophils not always occur in a primed state and are therefore less suitable for such toxicological studies.

## Abbreviations

APC: allophycocyanin
ATCC: American Type Culture Collection
ATRA: all-trans retinoic acid
CD: cluster of differentiation
CEACAM: carcinoembryonic antigen-related adhesion molecule
CNP: carbon nanoparticles
COPD: chronic obstructive pulmonary disease
DAPI: 4′,6-diamidin-2-phenylindol
DCF: dichlorofluorescein
DMF: dimethylformamide
DMSO: dimethyl sulfoxide
DNA: deoxyribonucleic acid
FACS: fluorescent-activated cell sorting
FCS: fetal calf serum
FITC: fluorescein isothiocyanate
GM-CSF: granulocyte-macrophage colony-stimulating factor
H_2_DCFDA: 2’,7’-dichlorodihydrofluorescein diacetate
Ig: immunoglobulin
MFI: mean fluorescence intensity
OPA: opsonophagocytosis assay
PAA: polyacrylic acid
PBS: phosphate buffered saline
PE: phycoerythrin
PerCP/Cy5.5: peridinin chlorophyll/cyanine5.5
PI: propidium iodide
PI3-K: phosphoinositide 3-kinase
PMN: polymorphonuclear neutrophils
ROS: reactive oxygen species
RPMI: Roswell Park Memorial Institute
SEM: standard error of the mean
